# Technical Requirements, Design, and Automation Process for a Statewide Registry-Based Tailored Text Messaging System: Protocol for a Longitudinal Observational Study

**DOI:** 10.2196/62874

**Published:** 2025-04-18

**Authors:** Deborah Ogunsanmi, Jerica Chambers, Asos Mahmood, Avinash Reddy Pakker, Anusha Kompalli, Umar Kabir, Satya Surbhi, Justin Gatwood, Md Sultan Mahmud, James E Bailey

**Affiliations:** 1 College of Graduate Health Sciences University of Tennessee Health Science Center Memphis, TN United States; 2 Center for Health System Improvement College of Medicine University of Tennessee Health Science Center Memphis, TN United States; 3 Tennessee Population Health Consortium University of Tennessee Health Science Center Memphis, TN United States; 4 Division of General Internal Medicine Department of Medicine University of Tennessee Health Science Center Memphis, TN United States; 5 College of Pharmacy University of Tennessee Health Science Center Memphis, TN United States; 6 Department of Preventive Medicine University of Tennessee Health Science Center Memphis, TN United States

**Keywords:** text messaging, clinical informatics, chronic disease, telehealth, telemedicine, electronic health records

## Abstract

**Background:**

Tailored text messaging is a low-cost mobile health intervention approach shown to effectively improve self-care behaviors and clinical outcomes for patients with chronic cardiometabolic conditions. Given the ubiquitous nature of mobile phones, text messages have the potential to reach a large audience. However, automating and disseminating tailored text messages to large populations at low cost presents major logistical challenges that serve as barriers to implementation.

**Objective:**

This study aimed to describe the protocol for a longitudinal observational study designed to assess the feasibility of an innovative approach for automating and disseminating personalized and tailored text messages to large populations at risk of cardiovascular events using a low-cost registry-based tailored text messaging system known as the Heart Health Messages (HHM) program. Further, it describes the technical requirements, architectural design, automation process, and challenges associated with program implementation.

**Methods:**

Patients at high risk of cardiovascular diseases are identified using a statewide population health registry known as the Tennessee Population Data Network. Tailored invitation messages and enrollment surveys are sent to eligible patients via Twilio. Upon completion of the receipt of consent and enrollment forms, participants receive tailored text messages from a library of generic messages based on participant-selected frequency of message delivery (daily or every other day). In addition, participants receive monthly text-based check-in survey messages designed to assess intervention adherence and improvement in self-care. Participants are also sent quarterly follow-up surveys to update enrollment information and preferences. All enrolled participants will receive tailored text messages for a 12-month intervention period.

**Results:**

Since the start of the program, 18,974 patients from 2 major health systems have met the inclusion criteria and were eligible for the HHM program. A total of 3 phases of HHM 1.0 have been implemented so far, reaching 225 eligible patients in phase 1, a total of 5288 patients in phase 2, and 13,461 patients in phase 3, with an enrollment of approximately 2% (n=4/225), 3% (n=137/5228), and 3% (n=350/13461), respectively. Efforts are underway to implement strategies in collaboration with the health systems to enhance the HHM program rollout and patient participation.

**Conclusions:**

The HHM program is a low-cost tailored text messaging intervention set for broader dissemination and potential replication. The program has the capacity to improve outcomes for people with chronic medical conditions.

**International Registered Report Identifier (IRRID):**

DERR1-10.2196/62874

## Introduction

### Background

The dramatic increase in the prevalence of obesity-associated chronic conditions, including hyperlipidemia, diabetes, and cardiovascular disease in the United States [1,2], demands a strong clinical and public health response. Given their low costs and high dissemination potential, mobile health interventions hold promise for promoting behavioral change and improving outcomes for patients with chronic conditions. Tailored motivational text messages have particular promise as extensive research demonstrates their effectiveness in encouraging healthy eating, physical activity, weight loss, management of chronic diseases, and medication adherence [3-8]. Motivational health-related text messages are text messages designed to support and encourage people to change specific health behaviors, such as healthy eating, exercising, or quitting smoking [9,10]. Tailored text message automation and dissemination to large populations at low cost remains logistically difficult. Many research groups have used population health registries to track and address gaps in care for people with diabetes and other cardiovascular conditions [11-13]. However, to our knowledge, no organizations have used registry-based data to populate a tailored motivational text messaging system.

This study describes the protocol for an innovative approach for automating and disseminating tailored text messages to large populations at low cost through a registry-based tailored text messaging system known as the Heart Health Messages (HHM) program. The HHM uses both patient input and a statewide population health registry [14] to tailor and personalize health-related text messages to patients identified at high risk for cardiovascular events. The HHM program is being made available to primary care practices through the Tennessee Heart Health Network (TN-HHN), a statewide quality improvement cooperative which seeks to implement evidence-based patient-centered interventions to improve cardiovascular care [15]. The current pilot study assesses the feasibility of the HHM program’s innovative approach for automating and disseminating personalized and tailored text messages to large populations with or at risk of cardiovascular disease across Tennessee.

### Research Context and Early Development of the HHM Program

The HHM program is a theory-based tailored text messaging platform initially developed for the Patient-Centered Outcomes Research Institute-funded Management of Diabetes in Everyday Life (MODEL) Study, a pragmatic randomized trial comparing the effectiveness of motivational text messaging with health coaching and educational materials for African American patients with uncontrolled diabetes [16,17]. The message library was created using patient-centered design with input from health behavior research, motivational interviewing, and patient experts. Messages focused on improving self-care behaviors related to healthy eating, exercise, and medication adherence were tailored using patient enrollment survey responses and periodic text message check-ins and were delivered using manual processes [18].

Our earlier research demonstrated that the MODEL Study tailored motivational text messaging program was highly effective in improving healthy eating and physical activity self-care behaviors for patients with or at high risk of cardiovascular disease. For example, MODEL patients who received both a health education toolkit and motivational text messages reported a significantly greater increase in the number of days following a healthful eating plan, with an average increase of 1.36 days per week (95% CI 1.07-1.64), compared with those who received only the health education toolkit who saw an average increase of 0.67 days per week (95% CI 0.30-1.04). Patients randomized to the motivational text messages intervention also significantly improved their blood sugar control. In addition, tailored text messages were found to be more effective in improving physical activity among suburban or rural populations compared with patients who received the education toolkit alone [17,19].

In preparation for the TN-HHN initiative, our regional population health data registry [14] was expanded statewide as the Tennessee Population Data Network (TN-POPnet) to support practice-level quality reporting, outcome tracking, and the HHM program [20]. The new HHM text messaging system sought to modify the MODEL motivational text messaging program to reduce dependence on the enrollment survey, increase the use of registry electronic health record data for tailoring, and automate the tailoring process and delivery of motivational text messages for statewide scaling and dissemination. The current pilot study also seeks to describe the process of scaling and automating the MODEL motivational text messaging program as a statewide registry-based tailored text messaging system and the technical requirements, architectural design, automation process, and challenges associated with program implementation.

## Methods

### Study Design

The HMM Pilot Study is a pragmatic pilot feasibility study that uses a prospective cross-sectional design with repeated measures (ie, longitudinal observational) to assess the participation experience of successive waves of participants recruited over time in the setting of evolving HHM program recruitment strategies informed by previous experience using a Plan-Do-Study-Act (PDSA) framework for continuous quality improvement [21]. Reporting for the HHM program description was based on the TIDieR (Template for Intervention Description and Replication) checklist. (Multimedia Appendix 1) [22].

### Study Setting

The initial piloting of the HHM program is being conducted in 2 major nonprofit health systems participating in the TN-HHN, including 1 regional safety net hospital system and 3 of its outpatient clinics and 1 large federally qualified health center with 7 major outpatient locations, in Memphis, Shelby County of West Tennessee.

### Participants and Eligibility Criteria

The HHM pilot study included all adults meeting one or more of the following criteria placing them at increased risk of cardiovascular disease; (1) diagnosis of one or more of the following conditions: obesity, diabetes, prediabetes, hypertension, hyperlipidemia, arrhythmia, and congestive heart failure requiring one or more prescription medication; (2) heart attack, hospitalization for chest pain, or stroke in the past 6 months; and (3) cardiac bypass surgery or cardiac catheterization. Patients were excluded if pregnant or currently taking medication for cancer, HIV, or viral hepatitis.

### Recruitment Procedures

Patients meeting the above inclusion criteria at high risk for cardiovascular events were identified using electronic health record data in the TN-POPnet. The eligibility criteria are applied to all adults seen in participating health system outpatient clinics within the past year. Patient identifiers were collected and used to send personalized invitation messages and enrollment surveys to all those meeting eligibility criteria.

### Heart Health Messages Program Technical Requirements

Before starting development, the following HHM program technical requirements and their proposed solutions were identified.

#### Secure Relational Database

The HHM required a secure relational database that meets the 1996 HIPAA (Health Insurance Portability and Accountability Act) requirements (ie, is HIPAA-compliant). TN-POPnet data, including direct patient identifiers, demographics, diagnoses, and laboratory values, were already housed in a fully HIPAA-compliant Oracle database. The database also includes patient-reported data, text message stems, schedule tables, and procedures and functions for tailoring and delivering text messages.

#### Secure Web-Based Application

The HHM required a HIPAA-compliant web-based application to capture patient-reported message preferences and health goals, beliefs, attitudes, and self-care motivation and activities [18]. Since REDCap (Research Electronic Data Capture; Vanderbilt University) [23] was used for MODEL, this platform was selected for HHM candidate consent and enrollment survey data collection and will be used for research purposes in program evaluation. Previous research studies have used REDCap for data capture and storage [24,25], and its compliance with HIPAA regulations have also been well described [23,26].

#### Short Messaging Service Gateway

The HHM required a HIPAA-compliant SMS gateway capable of sending text messages and receiving participant responses to periodic check-in messages. Based on extensive vetting of alternatives, Twilio (Twilio Inc) was selected as the SMS gateway and customer engagement communication platform [27].

#### Application Program Interface Services

The HHM required application program interface (API) services to connect and integrate REDCap, Twilio, and the Oracle Database. Since these applications all require use of representational state transfer (REST) API services, we used REST API services to query, monitor, tailor, and deliver personalized text messages [28].

### Development of HHM Content and Features

**Figure 1:**
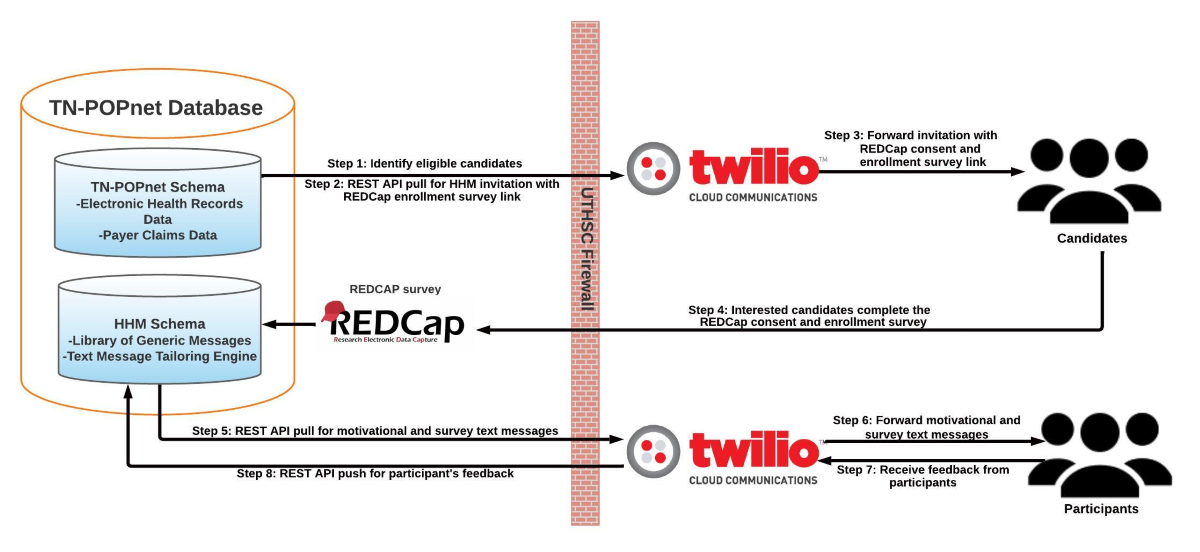
Heart health messages program architectural design. API: application program interface; HHM: heart health messages; REDCap: Research Electronic Data Capture; REST: representational state transfer; TN-POPnet: Tennessee population health data network.

Key HHM program content and features were adapted from the MODEL program system [18], including a text message library and a REDCap web-based data capture system, however, all features required additional development to suit the HHM program. Several alternative vendors were considered for each feature. Twilio was selected as the SMS gateway due to its ease-of-use API and Studio Flow tools. We initially considered Python (Python Software Foundation), MySQL (Oracle Corporation), and PHP (Zend Technologies) for text message personalization and tailoring but selected Oracle Procedural Language and SQL due to its ready integration into Oracle using Oracle Application Express. Existing MODEL program text message stems were then migrated into the HHM schema of the TN-POPnet Oracle database (Figure 1). Example stems are shown in Table 1.

**Table 1:** Example message stems used for the heart health messages program.

				Example message stems (ie, without personalization)
**Healthy eating focus**				
	**Frame**			
		Education	{First name}, grocery shopping tip: most fresh food is located along the walls of grocery stores. Try buying most of your food from these locations {First name}, before you buy any food, read its label. It has important info that will help you choose what foods are best for your blood sugar	
		Goal setting	{First name}, controlling diabetes can be challenging but there is a lot in your control, like reaching for your goal of healthy eating each day {First name}, eating healthier is a challenge for anyone but your health depends on it. Make eating healthy a goal and better health will be your reward	
		Motivation	{First name}, believing in the power of healthy foods is a winning attitude when you have diabetes. Turn that attitude into action and eat well all day {First name}, not everything about diabetes is easy. Eating healthy is a good first step in managing your care	
**Exercise focus**				
	**Frame**			
		Education	First name}, now that you have a workout goal, think about why you chose it. Thinking about why you exercise will make it easier to do each day {First name}, we know you are busy. But every step counts so do as much exercise as you can each day and add more time little by little	
		Goal setting	{First name}, you are on the way to being able to handle your diabetes. You may not be there yet, but every day you follow your exercise plan you get closer {First name}, taking a friend along for your workouts is a great way to stay healthy together and keep you on track to reach your physical activity goal	
		Motivation	{First name}, the hardest step is the first one. Once you get going on your physical activity plan each step you take will be easier. Start today! {First name}, handling your diabetes does not always happen overnight. Hang in there and follow your exercise plan, you wll be able to handle it better soon	
**Medication messages**				
	**Frame**			
		Education	{First name}, with {health condition} you have a higher risk of dying due to pneumonia. Talk with your doctor about whether you should get vaccinated. {First name}, every time you buy an over-the-counter medication be sure to check with your pharmacist, so you know it is safe to use with your {health condition} drugs.	
		Goal setting	{First name}, you may not believe it yet but you can take care of your {health condition}. Something to do each day to show it: meet your goal of taking your medications. {First name}, taking care of your {health condition} and taking your medicine can be tough, but you can do it. Ask your doctor about resources that may help you.	
		Motivation	{First name}, taking {#_of_prescribed_medications} medications is certainly a challenge. Know that each one is helping you improve your overall quality of life. {First name}, thinking you can meet the challenges of your {health condition} head on is a powerful attitude. Stay strong and keep up with your entire treatment.	
**Heart health focus**				
	**Frame**			
		Education	{First name}, {health condition} tends to lower your “good” cholesterol which means you need to stick to your treatment plan to help your body stay healthy. {First name}, trying to control {health condition} can be stressful. Do not let it get your down. Stick with your plan and the results will help with the stress.	
		Goal setting	{First name}, having {health condition} is nothing to be embarrassed about- it is just like any other condition that requires managing. Focus on you and your goals. {First name}, {health condition} can lead to poor blood flow in your legs. Having goals like eating well, exercising, and taking your medications will help prevent this.	
		Goal progress^a^	{First name}, your blood pressure is looking better, which means you are on the path toward even better health! {First name}, your “bad” cholesterol is on its way down! Keep sticking with your treatment plan and even better results on are the way {First name}, you are at a healthy weight for your height. Maintaining a healthy weight goes a long way to keep your heart healthy, too	
		Motivation	{First name}, we know that handling {health condition} is time consuming. Once it becomes habit I bet you'll see that it will just fit right in with your lifestyle. {First name}, treating {health condition} takes a team, including friends and family. Maybe they can make changes with you? Just ask. Their help can go a long way.	

^a^“Goal Progress” messages are tailored based on positive or negative changes in blood pressure, BMI, or cholesterol values.

MODEL schedule tables for sending messages based on participant message preferences (eg, for each combination of focus, frame, and frequency) were adapted for patients with and those without diabetes and migrated to the HHM schema. As shown in Table 1, the message stem, “focus,” represents the health behaviors participants would like to prioritize, such as healthy eating, physical activity, or medication adherence. The frame refers to the content and tone of the messages, which can be educational, motivational, or goal-oriented. The frequency determines the options for message delivery, including once a day or once every other day. Variables from REDCap enrollment and quarterly follow-up surveys were linked to the text message library in the HHM schema using APIs. Text-based monthly check-in surveys are initiated, and valid text responses are collected through Twilio Studio Flow and sent to the HHM schema through a HTTP request.

### Architectural Design of the HHM Program

HHM functionality requires electronic health records and patient-reported data collection, data analysis, message tailoring, and message delivery processes. The HHM program’s modular design allows each component to function independently and govern a specific system activity (Figure 1).

The HHM automated text messaging system relies on the TN-POPnet database (Figure 1). In step 1, SQL scripts apply predefined eligibility criteria to identify eligible patient candidates in TN-POPnet. In step 2, candidate identifiers (name, cell phone number, zip code, date of birth, and clinic information) are collected (REST API pull) from the TN-POPnet to personalize both invitation messages and enrollment surveys. Enrollment surveys are personalized by automatically feeding candidate information into the HHM schema using an SQL query to reduce candidate data entry time. In step 3, individualized invitation messages to the HHM program including a REDCap consent and enrollment survey link are forwarded to candidates’ mobile phones via Twilio. In step 4, interested candidates follow the REDCap link to consent, fill out the enrollment survey, and receive the HHM welcome message. Nonconsenting candidates automatically stop getting text messages and are given the option to receive another invitation in the future. Consenting candidates can also text “STOP” to halt further messages. In the case of no response, eligible candidates are resent the REDCap link to the HHM program after 2 days from the initial invitation, and this process is repeated after another 2 days. After that, participants will no longer receive an invitation. Consent forms and enrollment on the survey data are stored in REDCap and API-pulled into the HHM schema. In step 5, messages are tailored using the TN-POPnet registry data as well as data from the enrollment surveys. The HHM Schema makes an HTTP request to Twilio to prepare for sending tailored text messages and surveys.

In step 6, participants are forwarded tailored text messages from a library of generic messages via Twilio based on the selected frequency of message delivery (daily or every other day). Also, participants receive monthly text-based check-in survey messages (Textbox 1) designed to assess adherence and improvement in self-care based on participant-selected focus. Furthermore, participants are sent REDCap links to quarterly follow-up surveys to update enrollment information and focus, frame, and frequency preferences.

**Example monthly survey conversation between the heart health messages program and an anonymous participant (Jane).**

**HHM:** Jane, on how many of the last 7 days did you follow a healthy eating plan? Please REPLY with a number from 0 to 7.

**Jane:** Five

**HHM:** Sorry, we do not recognize the value of the answer. Please try again.

**HHM:** Jane, on how many of the last 7 days did you follow a healthy eating plan? Please REPLY with a number from 0 to 7.

**Jane:** 5

**HHM:** Thank you, you have completed the survey!

In steps 7 and 8, participant responses from the monthly check-in surveys and quarterly follow-up surveys are sent to the HHM schema via an API Push. Each combination of focus, frame, and frequency (n=27) has 3 buckets (A, B, and C) in which the majority of messages pertain to the participant’s selected focus, mixed with messages from the other 2 focus areas, with decreasing percentage of messages from nonselected foci, as described in our formative work [18]. Participants are changed to a different bucket (ie, mix of messages) when participant responses to the monthly check-in survey differ from their baseline enrollment surveys. Participants continue to receive messages from the same bucket for the next month when their responses are unchanged. Responses from the quarterly follow-up surveys are also used to update enrollment information, as discussed in step 5. If participants do not complete quarterly follow-up surveys or choose to remain in the same focus as the last quarter, they receive another combination of their previous quarter’s preference, referred to as an “instance.” The timing at which tailored text messages and all surveys are sent is controlled by the Oracle Job Scheduler module. Interested candidates complete the HHM program over a 12-month period or depending on the agreement with the patients’ health system or primary care practice.

### Patient-Centered Design of the HHM Program

**Figure 2:**
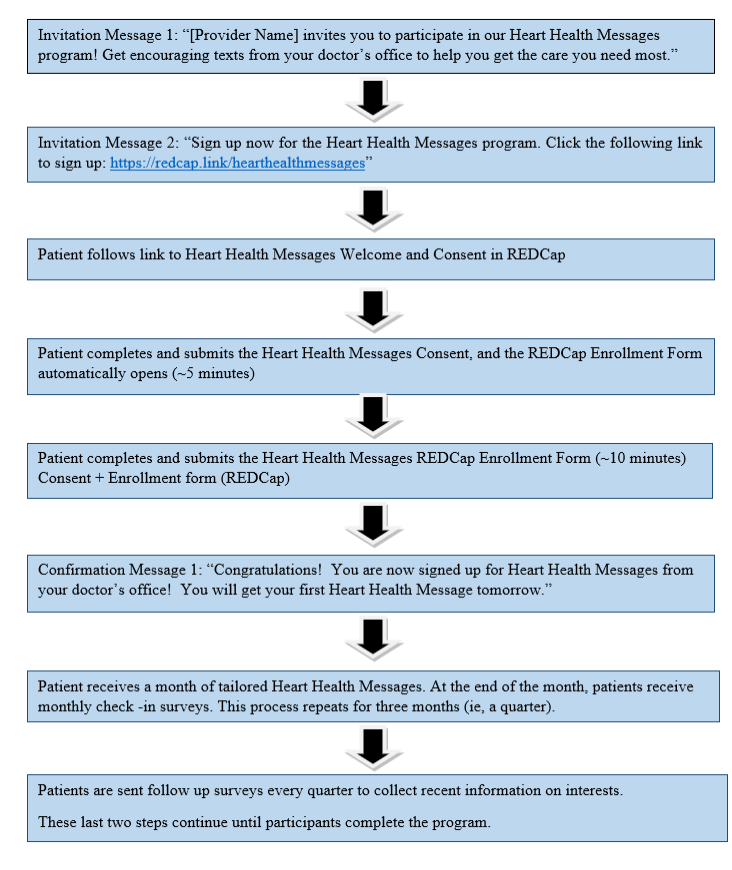
Patient experience flowchart illustrating the chronology of participant interactions with the heart health messages program. REDCap: Research Electronic Data Capture.

Throughout, HHM development was informed heavily by patient-centered design principles [29] to enhance patient experience of the HHM program (Figure 2). Before formal piloting of the system, we received feedback on system usability from 23 physicians and staff members volunteers, including some individuals with similar demographics and educational background to the target population. Feedback was used to make changes in system properties to improve user experience, including (1) shortening the enrollment survey to reduce respondent burden, (2) revising the consent form to use more patient-friendly language, (3) updating the invitation message to be more engaging, and (4) redesigning the enrollment workflow by dividing it into 2parts: a separate consent process followed by the baseline survey.

### Outcomes

The current ongoing pilot study process evaluation for all participating health systems and practices includes ongoing assessment of the number and proportion of (1) eligible patients consented, enrolled, and with incomplete enrollment tracking completed signups on a monthly basis for a 3-month sign-up period until outreach efforts are completed; (2) enrolled patients responding (ie, engaged) to monthly text-based check-in surveys; and (3) participant retention rates at 3 and 6 months.

The long-term HHM program outcomes evaluation will use a quasi-experimental study design using a difference-in-differences methodology and non-enrolled matched controls to evaluate the impact of the HHM program on major health outcomes including systolic and diastolic blood pressure, hemoglobin A_1c_, cholesterol, and BMI. The Difference-in-Differences approach is considered the most reliable advanced statistical method for program evaluation and assessment of the impact of non-randomized interventions, especially in primary health care settings [30,31]. The TN-POPnet registry, where patient and program measures and metrics are recorded, will also substantially support and facilitate the planned outcomes evaluation.

### Ethical Considerations

This study was approved by the institutional review board at the University of Tennessee Health Sciences Center (No. 21-08392-NHSR). All participants provided informed consent before their enrollment in the HHM program, and this consent was stored in our Secure Web-based Application REDCap. Patient participation was completely voluntary, and no compensation was involved.

## Results

So far, 18,974 patients in the 2 major health systems participating in the TN-HHN were eligible and included in the pilot study of the automated registry-based version of the tailored text messaging system (HHM 1.0). Eligible candidates will receive between approximately 212 and 428 messages based on selected message preference at enrollment. These messages cost US $0.008 each, and the estimated cost of sending a tailored text message per patient via Twilio for 12 months ranges from US $1.70 to US $3.42.

Table 2 shows the baseline results from the initial pilot cohort of HHM 1.0. In phase 1 (November 16-20, 2022), 225 eligible patients were sent invitation messages. Approximately 2% (4/225) completed the HHM consent and baseline survey following receipt of a text message invitation alone. In phase 2 (March 2-6, 2023), 5288 eligible patients were contacted, and approximately 3% (137/5228) responded and completed the HHM consent and enrollment survey**.** In phase 3 (September 22-26, 2023), invitations were sent to an additional 13,461 eligible patients and a 3% (350/13461) enrollment rate was observed. All consented and enrolled participants will receive tailored text messages for a 12-month period. Efforts are underway to implement strategies in collaboration with health systems to enhance the HHM program rollout and patient participation.

**Table 2:** Preliminary results from the initial pilot cohort of heart health messages 1.0 patients.

		Eligible and invited patients, n
**First phase of piloting (n=225)**		
	Did not respond to consent survey	218
	Did not respond to consent survey, stopped	0
	Completed consent survey, did not consent	2
	Completed consent survey, consented, did not complete baseline	1
	Completed consent survey, consented, stopped	0
	Consented, completed baseline survey, set to receive messages	4
**Second phase of piloting (n=5288)**		
	Did not respond to consent survey	4679
	Did not respond to consent survey, stopped	131
	Completed consent survey, did not consent	13
	Completed consent survey, consented, did not complete baseline	327
	Completed consent survey, consented, stopped	1
	Consented, completed baseline survey, set to receive messages	137
**Third phase of piloting (n=13,461)**		
	Did not respond to consent survey	12,536
	Did not respond to consent survey, stopped	0
	Completed consent survey, did not consent	34
	Completed consent survey, consented, did not complete baseline	540
	Completed consent survey, consented, stopped	1
	Consented, completed baseline survey, set to receive messages	350

## Discussion

### Anticipated Findings

The HHM program approach for using data from a statewide population health registry (ie, TN-POPnet) as well as patient input to tailor and personalize health-related text messages is highly innovative. To our knowledge, the HHM approach to registry-based message tailoring has not been previously described. This approach allows health systems to send tailored and personalized messages to large populations of patients at high risk for cardiovascular disease at a significantly lower cost compared with alternative interventions such as in-person counseling, traditional mail, or phone calls. These alternative traditional methods are usually more expensive and require intensive manpower labor [32-34] compared with the tailored text messaging approach, which is more cost-effective and scalable [35], particularly in rural and underserved populations where access to care is limited [36]. Another key strength of the HHM is its patient-centeredness. The HHM message library was established using a patient-centered design with critical input from health behaviors literature and feedback from patient experts [29]. HHM program participants are enabled to select and prioritize their own health goals (ie, healthy eating, physical activity, or medication adherence) at enrollment, and they can update enrollment information and focus, frame, and frequency preferences of incoming text messages during quarterly follow-up surveys. Further, participants are assessed monthly for program adherence and improvements in self-care. An additional strength of the HHM program is its ability to provide personalized and tailored progress feedback for some health outcome metrics (eg, blood pressure, cholesterol, and BMI) if they prioritize receiving “Goal Progress” messages, either at initial enrolment or at follow-up. Finally, the HHM is evidence-based; the nonautomated version of the text messaging program has already been tested and evaluated in the real world and demonstrated to change health behaviors in patients at increased risk for cardiovascular events **[17,19,37].**

**The low participation rate seen in HHM 1.0 is** consistent with other research demonstrating an average of approximately 3% response rates for unsolicited text messages [38]. However, previous research has also demonstrated that participation rates in similar text messaging programs can reach 20% or more with more intensive recruitment supported by the physician and other primary care team members [39]. The low enrollment rates observed could negatively impact the cost-effectiveness of the program as a large amount of text messages are sent to a relatively small number of participants, which could affect the overall cost-benefit ratio of the program. Although the cost of text messages is low, at US $0.008 each, the fixed administrative costs could potentially make it difficult for current participating health systems to invest significant resources unless higher program enrollment and effectiveness can be achieved. To increase participation rates and program effectiveness, we are implementing several strategies, including distributing posters, flyers, and patient letters and providing staff members support to encourage patient sign-ups at participating health systems. These strategies ensure the program reaches its full potential with respect to cost-effectiveness and improving patient health outcomes. Further piloting will focus on deploying this service to additional TN-HHN primary care practices serving eligible patients with or at risk of chronic disease across Tennessee. In accordance with Agile implementation methodology principles, ongoing piloting of the HHM program in new health systems and practices will be guided by the experience of initial pilot study participants [40].

Other states and organizations considering the use of a population health registry to tailor and send motivational text messages must also pay particular attention to patient privacy requirements. Despite strict HIPAA requirements regarding the transmission of Patient Health Information (PHI) via text messages, some bulk messaging services that claim to be HIPAA-compliant fail to provide secure connections outside their networks [41]. Moreover, US regulations regarding electronic communication through mobile technologies like SMS have no specific technological requirements regarding SMS security, which means any platform can be used to communicate with patients as long as it upholds a high level of patient security and privacy [42]. Thus, substantial due diligence is required to ensure the safe transmission of PHI via text message to ensure optimal security. Our HHM system’s technical and design requirements and architecture were carefully selected to ensure full HIPAA compliance while maintaining patient data confidentiality and security. Specifically, all PHI is stored in a HIPAA-compliant Oracle database-TN-POPnet database. REDCap, a secure web-based application designed for research purposes, was selected and used to collect consent and enrollment information. For messaging services, we selected Twilio (Twilio Inc), a HIPAA-compliant SMS gateway, to securely manage patient communication and protect patient data. In addition, we obtained consent from each patient before their enrollment into the program, and participants were thoroughly informed of the risks associated with transmitting PHI via text messages.

### Technical Challenges and Limitations

While developing the HHM program, we experienced some technical challenges and limitations. First, while the length of the enrollment survey was necessary for the proper tailoring of motivational text messages, it also served as a barrier to patient participation. We attempted to rectify this by reducing the length of the enrollment survey, but this still potentially served as a hurdle to enrollment. Future text messaging platforms should ideally seek to rely on EHR data and other data sources rather than requiring a lengthy survey to be filled out by patients or participants.

Second, there were technical difficulties with moving information in the MODEL program to our automated HHM program. While the medication table was relatively straightforward, the expansion of HHM to include more medical conditions increased complexity. Several MODEL stems explicitly dealt with diabetes, so these had to be changed to be more general or replaced with another stem from the same focus and frame.

Third, it is also possible that the use of outdated or incomplete registry data could present potential risks to patients, such as encouraging adherence to medications that have recently been discontinued. However, the HHM program sought to minimize these risks through the use of as close to real-time updates of its underlying TN-POPnet registry system. Fourth, the HHM system’s reliance on patient self-reporting during enrollment and follow-up surveys could impact the accuracy and reliability of the tailored messages sent to participants. However, the results obtained from the HHM system’s real-world experience in the MODEL program indicate a positive effect on health behaviors. Therefore, these potential accuracy and reliability issues might not have clinical significance. Fifth, some invitation messages were blocked by Twilio (ie, the carrier) as potential spam. Further program iterations will seek to overcome this difficulty through broad registration of the campaign. This means that the HHM program will be registered as an application-to-person Campaign with Twilio. Finally, scaling the HHM program across multiple health systems presents challenges, primarily related to the need for practice participation and data sharing with a third party. To address these challenges, we merged all data into a common data model (TN-POPnet database), which facilitates seamless integration and collaboration among participating practices. This also enables data standardization.

### Conclusion

The HHM program provides an innovative and low-cost solution for sending personalized and tailored motivational text messages on behalf of their primary care provider to patients at risk for cardiovascular events. The HHM approach for using both a statewide population health registry and patient input to tailor and personalize health-related text messages to patients at highest risk has high potential for broader dissemination and replication. The patient-centered design and architecture of HHM lends itself to program scalability and ongoing refinement to improve its cultural tailoring and incorporation of additional modules to address additional self-care behaviors linked to improved outcomes for people with common chronic conditions. The HHM Program also demonstrates the utility of statewide population health registries like the TN-POPnet, and how they can provide a strong foundation for the low-cost delivery of essential population health services that can enhance the management and outcomes of care for chronic conditions, particularly for underserved or low-resource communities. Studies are needed to further assess the impact of registry-tailored motivational text messaging on overall health care expenditures and support its implementation and dissemination in states nationwide.

## Appendix

Multimedia Appendix 1 TIDieR (Template for Intervention Description and Replication) checklist.

Multimedia Appendix 2 Example of motivational health-related text messages sent to participants, tailored to their selected “focus” and “frame”.

## Abbreviations

API: Application Program Interface
HHM: Heart Health Messages
HIPAA: Health Insurance Portability and Accountability Act
MODEL: Management of Diabetes in Everyday Life
PDSA: Plan-Do-Study-Act
PHI: Patient Health Information
REDCap: Research Electronic Data Capture
REST: Representational State Transfer
TIDieR: Template for Intervention Description and Replication
TN-HHN: Tennessee Heart Health Network
TN-POPnet: Tennessee Population Data Network

## Data Availability

The datasets generated or analyzed during this study are not publicly available due to privacy concerns but are available from the corresponding author on reasonable request.
