# Supplementary figures and images for: Technical Requirements, Design, and Automation Process for a Statewide Registry-Based Tailored Text Messaging System: Protocol for a Longitudinal Observational Study
Source: JMIR Res Protoc. 2025 Apr 18;14:e62874. doi: 10.2196/62874 (PMC12048783; doi:10.2196/62874)

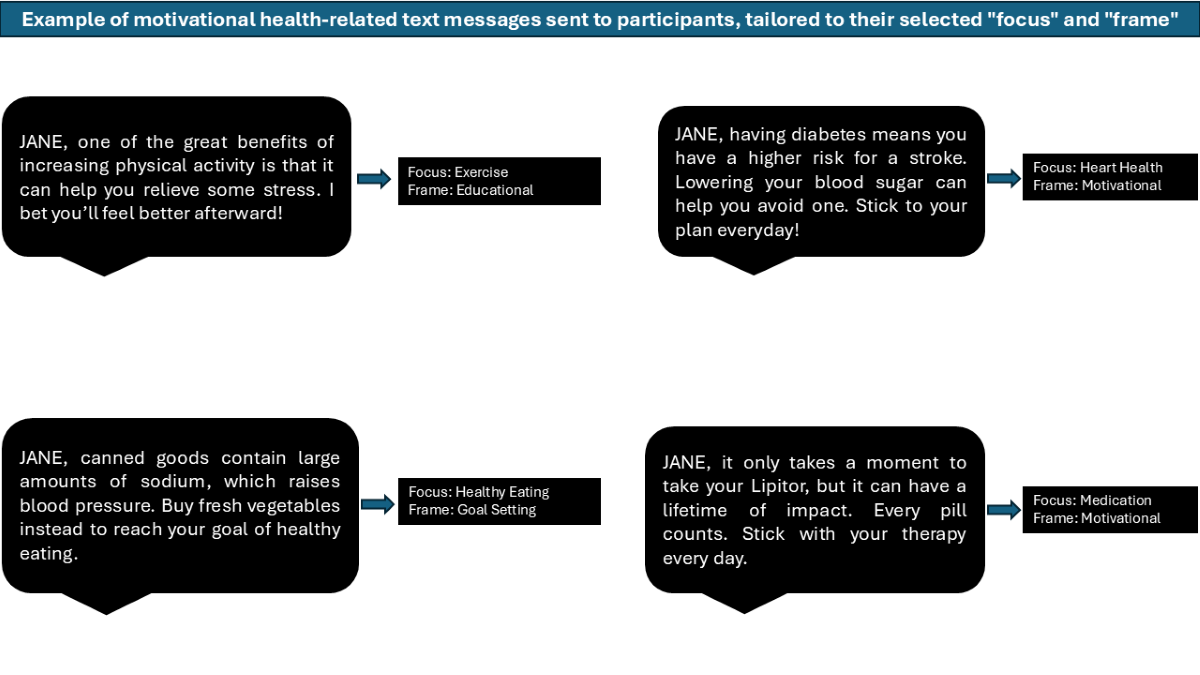

Supplement: Multimedia Appendix 2 [file resprot_v14i1e62874_app2.png]
